# A *Brassica* Exon Array for Whole-Transcript Gene Expression Profiling

**DOI:** 10.1371/journal.pone.0012812

**Published:** 2010-09-16

**Authors:** Christopher G. Love, Neil S. Graham, Seosamh Ó Lochlainn, Helen C. Bowen, Sean T. May, Philip J. White, Martin R. Broadley, John P. Hammond, Graham J. King

**Affiliations:** 1 Rothamsted Research, Harpenden, United Kingdom; 2 School of Biosciences, University of Nottingham, Sutton Bonington, Loughborough, United Kingdom; 3 Nottingham Arabidopsis Stock Centre (NASC), University of Nottingham, Sutton Bonington, Loughborough, United Kingdom; 4 Warwick HRI, University of Warwick, Wellesbourne, Warwick, United Kingdom; 5 Scottish Crop Research Institute, Invergowrie, Dundee, United Kingdom; United States Department of Agriculture, Agricultural Research Service, United States of America

## Abstract

Affymetrix GeneChip® arrays are used widely to study transcriptional changes in response to developmental and environmental stimuli. GeneChip® arrays comprise multiple 25-mer oligonucleotide probes per gene and retain certain advantages over direct sequencing. For plants, there are several public GeneChip® arrays whose probes are localised primarily in 3′ exons. Plant whole-transcript (WT) GeneChip® arrays are not yet publicly available, although WT resolution is needed to study complex crop genomes such as *Brassica*, which are typified by segmental duplications containing paralogous genes and/or allopolyploidy. Available sequence data were sampled from the *Brassica* A and C genomes, and 142,997 gene models identified. The assembled gene models were then used to establish a comprehensive public WT exon array for transcriptomics studies. The Affymetrix GeneChip® Brassica Exon 1.0 ST Array is a 5 µM feature size array, containing 2.4 million 25-base oligonucleotide probes representing 135,201 gene models, with 15 probes per gene distributed among exons. Discrimination of the gene models was based on an E-value cut-off of 1E^−5^, with ≤98% sequence identity. The 135 k Brassica Exon Array was validated by quantifying transcriptome differences between leaf and root tissue from a reference *Brassica rapa* line (R-o-18), and categorisation by Gene Ontologies (GO) based on gene orthology with *Arabidopsis thaliana*. Technical validation involved comparison of the exon array with a 60-mer array platform using the same starting RNA samples. The 135 k Brassica Exon Array is a robust platform. All data relating to the array design and probe identities are available in the public domain and are curated within the BrassEnsembl genome viewer at http://www.brassica.info/BrassEnsembl/index.html.

## Introduction

Microarrays are used widely in many organisms to study how the transcriptome varies during development, or in response to environmental perturbations or biotic challenges. Whilst direct sequencing of cDNAs may ultimately supplant microarray-based platforms for transcriptome analyses, microarrays retain advantages over next generation sequencing (NGS), including, (1) a wider dynamic range [1], [2], (2) the availability of robust normalisation and analysis techniques [3], (3) large public reference datasets [4]–[6], and (4) lower costs of performing a biologically replicated experiment; the advent of multiplex array platforms is likely to reduce these costs further.

The Affymetrix GeneChip® platform (Affymetrix, Santa Clara, CA, USA) is a widely used microarray technology in which each gene on the microarray is represented by multiple 25-mer oligonucleotide probes. GeneChip® arrays have been developed for a number of plants, including *Arabidopsis thaliana*, barley, *Brachypodium*, *Citrus*, cotton, grape, maize, *Medicago*, poplar, rice, soybean, sugarcane, tobacco, tomato and wheat (http://www.affymetrix.com/). The use of GeneChip® arrays for heterologous, or cross-species, transcriptome studies has extended the range of species for which transcriptomics experiments have been reported [7]–[10].

For transcriptome analyses, oligonucleotide probes on GeneChip® arrays have often been targeted to 3′ gene sequences. This is because sequence data are typically derived from EST sequence collections with a 3′ bias, and the 3′ end of genes are generally more variable, which provides greater specificity. In addition, for many years hybridisation probes have conventionally been labelled from the 3′ end. However, 3′-biased arrays do not allow exon level analysis of gene transcripts and their splice variants. In contrast, whole transcript (WT) and tiling arrays allow for exon level interrogation of transcripts and splice variants. The latest GeneChip® arrays have probes for every exon in the genome, which results in greater specificity and a more accurate measure of transcript abundance [11]. Exon GeneChip® arrays can also be used to detect alternate splicing [12], sequence polymorphisms [13], [14], and for deletion mapping [15]. Whilst high probe-density tiling array platforms have been developed for *A. thaliana* [16]–[18], to date no WT exon GeneChip® array has been publicly available for plants.

The aim of this study was to develop a WT exon GeneChip® array for *Brassica*. *Brassica* has a complex genome structure typical of many crop plants. A series of genome duplication events leading to the diploid species has resulted in most genes being present in multiple paralogous and homeologous copies, which is compounded in the allopolyploid species. The Brassicaceae are a model system for studying plant genome evolution [9]–[21]. The genus *Brassica* includes the closest crop relatives of *Arabidopsis thaliana*, with relatively recent hybridisation events between representatives of the diploid A- genome of *B. rapa* (vegetable and oil crops) and C- genome of *B. oleracea* (vegetable crops) giving rise to the widely grown amphidiploid *B. napus* (AC-genome; canola/oilseed rape/colza, rutabaga/swede). Extensive genomic resources for *Brassica* species have already been assembled and are available through the ongoing Multi-national Brassica Genome Project (MBGP; www.brassica.info). In addition to >1.9 m *Brassica* sequences in Genbank, the *B. rapa* genome sequence is being released in 2010, alongside other reference genome and re-sequencing projects http://www.brassica.info/resource/sequencing.php). Efforts to study genome evolution and to underpin crop improvement will therefore benefit from a robust WT exon GeneChip® array for transcriptomics.

## Materials and Methods

### Selection of unigenes

**Figure 1:**
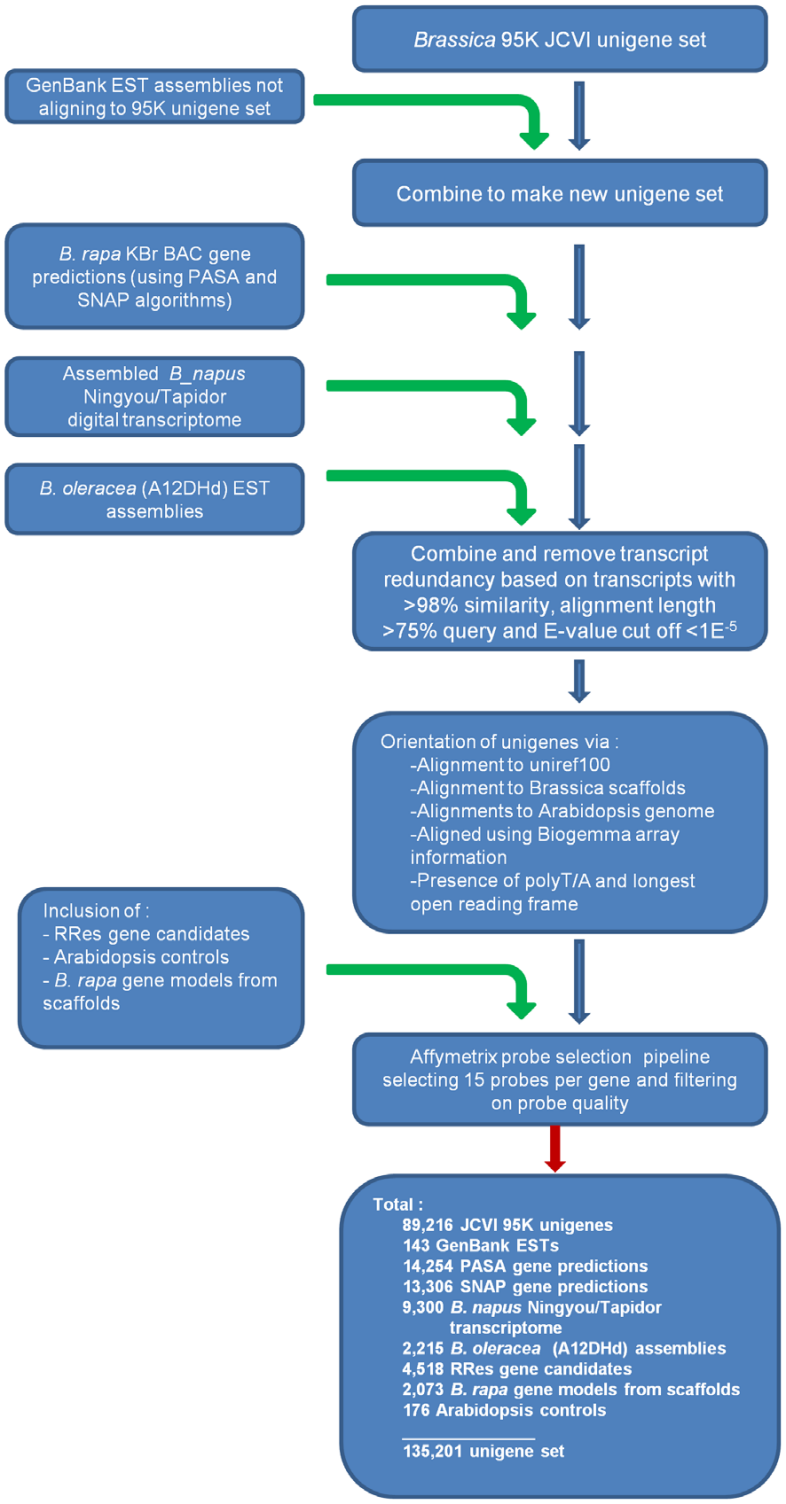
Work flow of the Affymetrix GeneChip® Brassica Exon 1.0 ST Array, data selection pipeline. Data were collated from several sources. Collated dataset were filtered to remove redundancy and orientated where possible. Unigenes passing the Affymetrix quality thresholds were tiled onto the array.

The pipeline leading to the final array design included an initial collation of *Brassica* gene model and transcript data available in December 2009. The source data used are summarised in Table 1. The starting point was a pre-existing Unigene set containing 94,558 sequences defined at the J. Craig Venter Institute (JCVI) in August 2007, which had been used to develop a 95 k oligo array based on 60-mers (95 k Brassica 60-mer array)[22]. Since this set was composed of assemblies of ESTs from different *Brassica* species, a detailed breakdown of unique genes by species is not possible. The dataset was processed through a number of filtering steps to avoid redundancy, and where possible to orientate transcripts in consistent 5′ to 3′ direction (Fig. 1). Additional transcriptome sequence datasets were added to the JCVI unigene set where they were deemed to be unrepresented. Datasets included 1,085 contigs formed from 2,122 assembled EST sequences downloaded from GenBank in May 2009. These reads were vector trimmed using CrossMatch (http://www.phrap.org/phredphrap/phrap.html) and sequences with a length of >100 bp were assembled using CAP3 [23](94% identity). A set of 7,434 *B. oleracea* (A12DHd) ESTs not present within GenBank at that date were also vector trimmed and assembled with parameters previously stated providing an additional 2,253 unigenes. Approximately 40 million Solexa (Illumina Inc., San Diego, CA, USA) sequenced ESTs were also included from the ‘digital transcriptome’ of *B. napus* lines TapidorDH and Ningyou7 [24], which was assembled using Velvet [25] with minimum contig length of 100 bp, coverage cut-off of five and k-mer value of 23, producing 29,956 contigs.

**Table 1:** Components of the Brassica 135 k unigene set.

Source	Number of gene models
JCVI unigene set not represented by gene predictions	89,216
PASA gene predictions from *B. rapa* KBr BACs	14,254
SNAP gene predictions from *B. rapa* KBr BACs	13,306
*Brassica napus* N/T digital transcriptome (Velvet) assemblies	9,300
*Arabidopsis thaliana* gene models	4,518
*B. oleracea* A12DHd EST assemblies (cap3 94%id)	2,215
*B. rapa* gene models from sequence scaffolds	2,073
ESTs not represented in JCVI unigene set extracted from Genbank on 14/05/09 (assembled with cap3 94% id)	142
*A. thaliana* controls	176
**Total set:**	**135,201**

Transcript redundancy within the combined datasets was eliminated based on empirically determined criteria, using BLAST [26]. Thus unigenes were eliminated where they aligned with >98% identity over >75% of the query sequence, with an Expect (E-) value <1E^−5^. This reduction in sequence redundancy resulted in a unigene dataset of 105,481 (Table 1).

The orientation of the combined unigene set was also established using defined criteria. Firstly, 76,687 unigenes were orientated by alignment using BLAST to Uniref100 [27], with E-value cut-off 1E^−5^. Those unigenes not aligning significantly to Uniref100 were aligned to *Brassica* genomic scaffolds and *A. thaliana* genomic sequence using the TimeLogic® GeneDetective™ algorithm (Active Motif Inc., Carlsbad, CA, USA), which orientated a further 36,170 unigenes. An additional 4,124 unigenes were orientated using available signal information from another Affymetrix GeneChip® array (courtesy of Biogemma, Paris, France) based on the 95 k JCVI unigene set. This enabled us to determine where the transcript generated a signal through hybridisation with *Brassica* cDNA, and so was in the correct orientation. In addition, the longest open reading frame was used to orientate a further 5,183, and the presence of a poly-A (Poly-T) tail was used to orientate 908 unigenes. The remaining 9,712 unigenes could not be orientated with confidence.

Predicted genes were also included in the array design, derived from 974 publicly available *B. rapa* Chiifu-401 KBr BAC sequences (BrGSP) using SNAP [28] and PASA [29] programs, with 15,817 and 17,558 genes identified respectively. A total of 4,913 unigenes were removed due to redundancy between the combined unigene set and predicted genes by the same criteria as previously, with preference for retention of the gene prediction over a redundant unigene alignment. An additional 2,073 gene models were included from preliminary annotation of *B. rapa* Chiifu-401 sequence scaffolds generated from high throughput sequencing. This sequencing project is led by Xiaowu Wang (Institute of Vegetables and Flowers, Chinese Academy of Agricultural Sciences, Beijing) who kindly provided pre-publication comparative analysis to identify gene models where these did not correspond to genes identified above. A subset of candidate *A. thaliana* gene models (4,517) that were not otherwise represented by *Brassica* orthologs were also included in the design. Three *Brassica* and 176 *A. thaliana* controls were also included within the design. The final unigene set available totalled 142,997 (Table 1).

### Array design

The selected Affymetrix GeneChip® format for the Brassica Exon 1.0 ST Array (135 k Brassica exon array) had capacity for 2.44 million 25 bp oligonucleotide probes of 5 µm (49-7875 format). The total unigene dataset was further filtered using the Affymetrix probe selection pipeline. Standard Affymetrix *A. thaliana* control and reporter sequences were added (89 genes). Probe sets were selected based on 15 probes per gene. In order to maximise the ability of the Affymetrix exon array to resolve paralogous genes which may differ at the exon level, and to detect alternative splicing, it was necessary to determine, where possible, the exon boundaries for each identified unigene or gene prediction. Since not all the unigenes had defined exon boundaries, this was achieved using the exon predictions derived from the TimeLogic® GeneDetective™ algorithm (Active Motif Inc.), where significant alignments (E-value<1E^−15^) existed between *A. thaliana* genomic sequence and *B. rapa* genomic scaffolds. Where exon boundary information could not be obtained, probes were evenly distributed over the length of the unigene.

In total, 1,043 unigenes were excluded as they did not pass quality filtering due to (1) the unigene being too small to design any probes of high enough quality, (2) potential probe cross-hybridisation, or (3) too low complexity. For 6,733 unigenes, probe sets could not be designed in a way that would distinguish them from other probe sets, and so these are not represented. In total there were 338,195 probe sets marked for tiling, containing a total of 2,416,447 probes that represented 135,201 unigenes.

### Plant growth and tissue preparation

Plants of the homozygous *B*. *rapa* line R-o-18 [30] were grown for 23 d in 13 cm diameter pots containing 1 L of an unmodified high-nutrient, peat-based substrate (Levington M3 Pot and Bedding Compost, Scotts Professional, UK; pH 5.3–5.7, N:P:K; 280∶160∶350 g m^−3^). Plants were grown under glasshouse conditions in May 2009 (16 hr photoperiod, 22.3°C and 13.3°C mean day and night temperatures respectively, irrigated with mains water). Two full leaves, including petioles and midribs, from three replicate plants, were harvested and frozen in liquid nitrogen. Root tissue samples were obtained from plants grown on agar plates. Surface sterilised seed were sown in large square (20×20 cm) tissue culture plates (QTray X6024, Genetix Ltd., New Milton, UK) containing 250 mL 0.8% agar (A1296, Sigma-Aldrich Company Ltd., Dorset, UK) and 1× MS salts (M5524, Sigma), adjusted to pH 5.6 with NaOH, under the conditions described previously [8]. Ten days after sowing, root tissue from 38 plants was pooled and snap-frozen at −70°C for each independent biological replicate.

### RNA preparation and hybrisation

RNA was extracted from tissue samples using a modified TRIzol extraction method [8]. Extracted total RNA was then purified using the ‘RNA Cleanup’ protocol for RNeasy columns with on-column DNase digestion to remove residual genomic DNA (Qiagen, Crawley, West Sussex, UK). Samples of total RNA were checked for integrity and quality using an Agilent Bioanalyser (Agilent Technologies, Santa Clara CA, USA). RNA samples were then split to allow the same RNA sample to be labelled and hybridised to the 135 K Brassica Exon array and the Agilent 95 k Brassica 60-mer array [22]. For the 135 K Brassica Exon array, RNA samples were labelled and hybridised according the manufacturer's instructions (Affymetrix, Santa Clara, CA, USA) at Nottingham Arabidopsis Stock Centre (NASC; http://affymetrix.arabidopsis.info). Briefly, 500 ng of total RNA from each sample was labelled using the Ambion WT expression kit (Ambion Inc, Austin, TX, USA). The end labelling, hybridisation, washing and scanning were performed according to the GeneChip® WT terminal labelling and hybridisation user manual (www.affymetrix.com), and scanned using an Affymetrix 3000 7G scanner. Following scanning, non-scaled RNA signal intensity files (.cel) were generated using the Command Console software (Affymetrix). Raw data are MIAME compliant as detailed on the MGED Society website http://www.mged.org/Workgroups/MIAME/miame.html and have been submitted to Gene Expression Omnibus (GEO; http://www.ncbi.nlm.nih.gov/projects/geo/; accessionGSE23141) and to NASC (http://arabidopsis.info/StockInfo?NASC_id=N9903). For the 95 k Brassica 60-mer array, RNA samples were labelled with the QuickAmp Labelling kit (Agilent Technologies) and hybridised to the array for 17 hours at 65°C at 10 rpm. The 95 k Brassica 60-mer arrays were washed, and then scanned on an Agilent G2565CA scanner, according to the manufacturer's instructions, and data files generated using Agilent Feature Extraction Software (version 10.7.3.1, Agilent Technologies). All raw data have been submitted to Gene Expression Omnibus (GEO; http://www.ncbi.nlm.nih.gov/projects/geo/; accession GSE23141.

### Data analysis

All data were analysed using GeneSpring GX (version 11.0.2, Agilent Technologies). For the 135 k Brassica Exon arrays, six RNA. cel files (three root and three leaf files) were normalized using the RMA pre-processor in GeneSpring GX. For the 95 k Brassica 60-mer array data files were imported into GeneSpring and a quantile normalization was applied. Normalized signal values for individual probes/probe-sets were standardized to the median signal value for the probe/probe-set within each array platform. All data were pre-filtered to remove genes whose normalized fold change was between 0.77 and 1.3 i.e. not changing. Genes with differential transcript abundance between leaf and root tissue were identified from the pre-filtered genes using a one-way ANOVA (GeneSpring) with a Benjamini-Hochberg corrected *p*-value <0.01 and a fold-change cut-off >2.

To enrich annotation of the probes and probe-sets, *A. thaliana* homologues were derived from the highest scoring alignment to *A. thaliana* coding sequences (TAIR v9)[31] using the TimeLogic® Tera-BlastN™ algorithm (Active Motif Inc.) with E-value cut-off at 1E^−5^. Arabidopsis gene descriptions and Gene Ontology (GO) annotation were obtained from TAIR (www.arabidopsis.org; TAIR genome v9, 11/06/2010). Identification and enrichment of GO terms within significantly differentially regulated sets of genes were obtained using the GO Browser function in GeneSpring GX with a Benjamini-Hochberg corrected *p*-value <0.05.

For alternate splicing analysis, the data were loaded into Genespring GX using the Affymetrix Exon splicing option, with the exon technology provided by Agilent Technologies. The data were normalised using the ExonRMA16 pre-processor and normalised signal values for individual probe-sets were standardised to the median value for the probe-set. Potential alternately spliced transcripts were identified by filtering on the splicing index (> = 5) and visualising them using the splicing visualization tool in Genespring GX. The splicing index for a probe-set is defined as the difference between gene normalized intensities for two chosen conditions.

Analysis the of 3′ bias in control probes was performed in Excel. The RMA-normalised signal values for 34 control probes (AFFX-BioB-5, AFFX-BioB-3, AFFX-BioC-5, AFFX-BioB-3, AFFX-BioDn-5, AFFX-BioDn-3, AFFX-CreX-5, AFFX-CreX-3, AFFX-DapX-5, AFFX-DapX-3, AFFX-LysX-5, AFFX-LysX-3, AFFX-PheX-5, AFFX-PheX-3, AFFX-ThrX-5, AFFX-ThrX-3, AFFX-TrpnX-5, AFFX-Trpn-3, AFFX-r2-Ec-bioB-5, AFFX-r2-Ec-bioB-3, AFFX-r2-Ec-bioC-5, AFFX-r2-Ec-bioC-3, AFFX-r2-Ec-bioD-5, AFFX-r2-Ec-bioD-3, AFFX-r2-P1-cre-5, AFFX-r2-P1-cre-3, AFFX-r2-Bs-dap-5, AFFX-r2-Bs-dap-3, AFFX-r2-Bs-lys-5, AFFX-r2-Bs-lys-3, AFFX-r2-Bs-phe-5, AFFX-r2-Bs-phe-3, AFFX-r2-Bs-thr-5, AFFX-r2-Bs-thr-3) were exported from Genespring for leaf and root from the Brassica exon and Arabidopsis experiments. The 3′ to 5′ ratio was calculated for each pair of probes for each gene and a one-tailed t-test was performed on the ratios.

The density plots were generated using the density function in the freely available statistical package R (version 2.9.2), using mean RMA-normalised signal values from leaf and root samples hybridised to the Affymetrix Brassica Exon 1.0 St array and the 95 k Brassica 60-mer array.

## Results and Discussion

**Figure 2:**
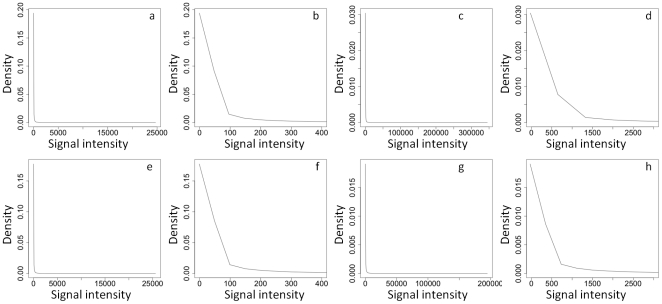
Dynamic range of probe set signals. Density plots of (a, b, e, f) mean probe-set signals from the 135 k exon array, and (c, d, g, h) mean probe signal values from the 95 k 60-mer array, for (a, b, c, d) leaf and (e, f, g, h) root tissue of *Brassica rapa* R-o-18 (n = 3).

**Figure 3:**
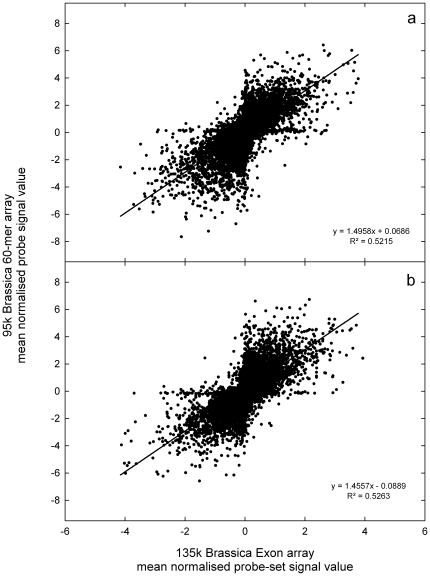
Comparison of transcript abundance on different array platforms. Relationship between mean normalised probe-set signals from the 135 k Brassica Exon array and mean normalised probe signal values from the 95 k Brassica 60-mer array, for a) leaf and b) root tissue of *Brassica rapa* R-o-18 (n = 3). Relationships between probe-sets from the 135 k Brassica Exon array and probes from the 95 k Brassica 60-mer array are based on shared *Arabidopsis thaliana* gene models.

The probe selection process for the Affymetrix GeneChip® Brassica Exon 1.0 ST Array (135 k Brassica Exon array; Fig. 1) marked 338,195 probe sets for tiling, containing a total of 2,416,447 probes that represented 135,201 unigenes (Tables 1 and 2). All probe and design data are publicly available from Affymetrix. The distribution of mean probe-set signals from the 135 k Brassica Exon array has large dynamic range (Fig. 2) and detected 11,078 significantly differently expressed transcripts (*p*<0.01) between leaf and root samples. Overall, there was a good correlation in transcript abundance (r^2^>0.5) between platforms, based on shared homology to *A. thaliana* gene models (Fig. 3).

**Table 2:** Summary statistics for the probe set withing the Affymetrix GeneChip® Brassica Exon 1.0 ST Array.

Summary statistics	base pairs
Total base count	113,812,609
Mean length	842
Standard deviation	28
Maximum length	17,365
Minimum length	78

Comparison of the 3′ bias in the hybridisation of control probes between the Brassica exon array and an Arabidopsis experiment using the Affymetrix ATH1 array (leaf, 7 days old, ATGE_5 A–C, GEO accession GSE5630 and root, 7 days old, ATGE_3A–C GEO accession GSE5631)[32], showed that the bias was significantly greater in the ATH1 hybridisations (one-tailed T-Test, P = 0.022, n = 34). This demonstrates that the labelling protocol used for the exon array produces a more consistent signal across the whole transcript as compared to the 3′ bias seen with older labelling protocols.

Genes up and down regulated in leaves compared with roots were found to be highly similar between the two Brassica array platforms. Based on comparison with the Arabidopsis datasets described above, they were also were broadly similar with a published leaf vs root transcriptome comparison obtained using *A. thaliana*. Similarity was defined by *A. thaliana* GO categories common to the different platforms.

An earlier study comparing six different platforms for the mouse transcriptome suggest a good correlation (Pearson product-moment correlation  = 0.7) between Affymetrix and Agilent platforms [33]. A similar result was obtained between these platforms for Arabidopsis [34]. Studies based on extensive survey of many arrays indicate that not all probes within an exon correlate and some probes may appear as outliers. This may be due to a wide range of factors, including multiple polyadenylation sites, antisense expression, the sequence of the probes, position of the probe on the array [35]–[37]. Thus the identification and analysis of such outlier probes may be useful indicators for deteting novel biological properties.

**Figure 4:**
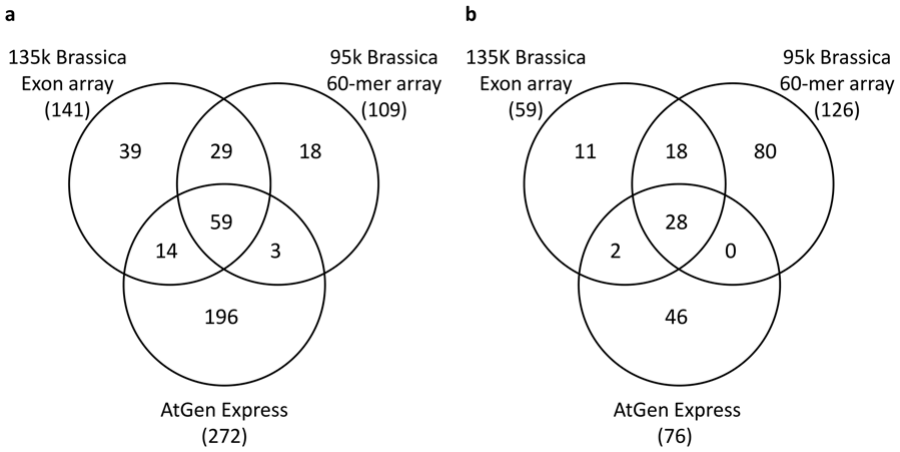
Gene Ontology categories of tissue-specific transcripts. Gene Ontology (GO) categories of overrepresented (p<0.05) genes whose transcript abundance was greater (a) or less (b) in leaves compared with roots of *Brassica rapa* R-o-18. GO categories are based on putative gene orthology between *Brassica* and *Arabidopsis thaliana* (TAIR v9). The three portions of each Venn figure represent the Affymetrix GeneChip® Brassica Exon 1.0 ST Array (135 k Brassica Exon array, n = 3), the Agilent 95 k Brassica 60-mer array (n = 3), and *A. thaliana* data from the AtGen Express data set for leaves (leaf, 7 days old, ATGE_5 A–C, GEO accession GSE5630) and roots (root, 7 days old, ATGE_3A–C, GEO accession GSE5631; Schmid et al., 2005).

Among genes where transcript abundance, detected by the 135 k Brassica Exon array, was greater in leaves compared to roots 141 GO categories were significantly (Benjamini and Hochberg (BH) corrected *p*<0.05) over-represented (Fig. 4a). Among these, 88 of the GO categories were in common with GO categories overrepresented on the 95 k Brassica 60-mer array platform (out of a total of 126 GO categories identified as significantly (BH corrected *p*<0.05) over-represented), and 73 GO categories were in common with GO categories overrepresented among genes whose transcript abundance was greater in leaves compared to roots in an experiment on *A. thaliana* (out of a total of 272 GO categories identified as significantly (BH corrected *p*<0.05) over-represented). Similarly, the 135 k Brassica Exon array detected 59 GO categories overrepresented (BH corrected *p*<0.05) among genes whose transcript abundance was less in leaves compared to roots (Fig. 4b). Among these, 46 of the GO categories were in common with GO categories overrepresented on the 95 k Brassica 60-mer array platform (out of a total of 126 GO categories identified as significantly (BH corrected *p*<0.05) over-represented), and 30 GO categories were in common with GO categories overrepresented among genes whose transcript abundance was less in leaves compared to roots in an experiment on *A. thaliana* (out of a total of 76 GO categories identified as significantly (BH corrected *p*<0.05) over-represented). As expected, GO categories identified as being significantly over-represented among genes whose transcript abundance was greater in leaves compared with roots were dominated by those associated with photosynthesis and chloroplasts (Table S1). For GO categories identified as being significantly over-represented among genes whose transcript abundance was less in leaves compared with roots, many were associated with responses to inorganic ions and abiotic stresses, consistent with roots role in the acquisition of water and mineral nutrients (Table S2).

**Figure 5:**
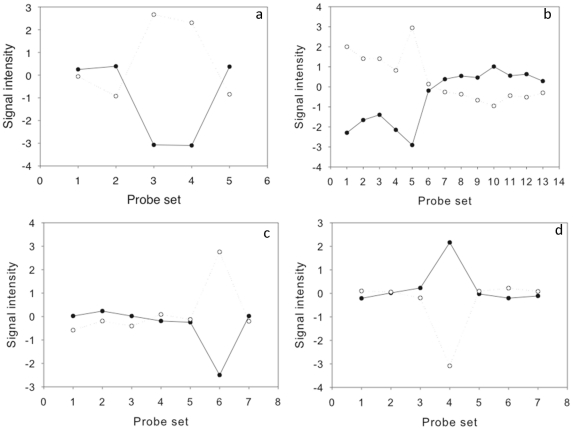
Potential alternatively spliced transcripts. Mean gene-normalised probe-set signals for leaf (open circle) and root tissue (closed circle) of four transcripts (a-rres037505, b-rres046838, c-rres107548, d-rres004182).

The design of the array should enable analysis of data at the exon level as well as the whole transcript level, in order to identify alternatively spliced transcripts. The 135 k Brassica Exon array has an average of 15 probes per gene, so there are a variable number of probes per exon, which may reduce the resolution of this analysis for some genes. However, preliminary analysis at the exon level indicates that the signal from each exon within a transcript is consistent, and that potentially alternately spliced transcripts can be identified (Fig. 5) using analysis by splicing index. Interestingly the Arabidopsis best BLAST hit of these four transcripts are also potentially alternatively spliced (as shown by the alternative splicing visualisation tool at the Plant DGB database; http://plantdbg.org/ASIP). These potentially alternatively spliced transcripts need to be confirmed experimentally to demonstrate the effectiveness of this array for alternative splicing analysis.

In conclusion, we describe the development of the Affymetrix GeneChip® Brassica Exon 1.0 ST Array. This is a 5 µM 49-7875 format array, containing 2.4 million 25-base oligonucleotide probes representing 135,201 gene models, with 15 probes per gene distributed among exons. The exon array is robust based on preliminary analyses of (1) dynamic range, (2) low CVs between biological replicates, (3) transcriptome differences between leaf and root tissue of a reference homozygous *Brassica rapa* line (R-o-18), according to overrepresented GO categories and technical comparison with an existing commercial array platform, (4) exon level data show that the majority of exons with a transcript have similar signal intensities and that potential alternatively spliced transcripts can be identified. Further analyses and validation will be facilitated in due course as additional datasets are released into the public domain, *sensu A. thaliana*. The 135 k unigene set is accessible as a track within the public BrassEnsembl genome browser at http://www.brassica.info/BrassEnsembl/index.html, and also as a Blast dataset within BrassEnsembl. In addition, the exon sequences, probeset and best hit alignments to Arabidopsis are available from http://www.brassica.info/resource/trancriptomics.php. It is anticipated that the Affymetrix GeneChip® Brassica Exon 1.0 ST Array will become a valuable tool for transcriptomics and mapping in several important crop species and will contribute to efforts to decipher genome evolution and adaptation within the Brassicaceae family.

## Supporting Information

Table S1 Common GO categories over-represented in genes whose transcript abundance is greater in leaves compared with roots of Brassica rapa R-o-18
(0.08 MB DOC)

Table S2 Common GO categories over-represented in genes whose transcript abundance is less in leaves compared with roots of Brassica rapa R-o-18
(0.04 MB DOC)
