# Supplementary material for: A Brassica Exon Array for Whole-Transcript Gene Expression Profiling
Source: PLoS One. 2010 Sep 16;5(9):e12812. doi: 10.1371/journal.pone.0012812 (PMC2940909; doi:10.1371/journal.pone.0012812)
Supplement: Table S1 — Common GO categories over-represented in genes whose transcript abundance is greater in leaves compared with roots of Brassica rapa R-o-18 (0.08 MB DOC) [file pone.0012812.s001.doc]

**Table S1** Common GO categories over-represented in genes whose transcript abundance is greater in leaves compared with roots of *Brassica rapa* R-o-18

| GO accession | GO Term | BH corrected *p*-value | | |
| --- | --- | --- | --- | --- |
|  |  | 135k Brassica Exon array | 95k Brassica 60-mer array | AtGen Express data |
| GO:0031976 | plastid thylakoid | 0 | 0 | 0 |
| GO:0031984 | organelle subcompartment | 0 | 0 | 0 |
| GO:0031975 | envelope | 0 | 0 | 0 |
| GO:0031967 | organelle envelope | 0 | 0 | 0 |
| GO:0031090 | organelle membrane | 0 | 0 | 0 |
| GO:0005737 | cytoplasm | 0 | 0 | 0 |
| GO:0034357 | photosynthetic membrane | 0 | 0 | 0 |
| GO:0005623 | cell | 0 | 0 | 1.76E-31 |
| GO:0005622 | intracellular | 0 | 0 | 0 |
| GO:0055035 | plastid thylakoid membrane | 0 | 0 | 0 |
| GO:0009941 | chloroplast envelope | 0 | 0 | 0 |
| GO:0042651 | thylakoid membrane | 0 | 0 | 0 |
| GO:0005575GO:0008372 | cellular component | 0 | 0 | 1.9E-32 |
| GO:0044464 | cell part | 0 | 0 | 1.76E-31 |
| GO:0044446 | intracellular organelle part | 0 | 0 | 0 |
| GO:0044444 | cytoplasmic part | 0 | 0 | 0 |
| GO:0043226 | organelle | 0 | 0 | 0 |
| GO:0043227 | membrane-bounded organelle | 0 | 0 | 0 |
| GO:0044436 | thylakoid part | 0 | 0 | 0 |
| GO:0043229 | intracellular organelle | 0 | 0 | 0 |
| GO:0043231 | intracellular membrane-bounded organelle | 0 | 0 | 0 |
| GO:0044435 | plastid part | 0 | 0 | 0 |
| GO:0044434 | chloroplast part | 0 | 0 | 0 |
| GO:0044424 | intracellular part | 0 | 0 | 0 |
| GO:0009507 | chloroplast | 0 | 0 | 0 |
| GO:0044422 | organelle part | 0 | 0 | 0 |
| GO:0009526 | plastid envelope | 0 | 0 | 0 |
| GO:0009532 | plastid stroma | 0 | 0 | 0 |
| GO:0009534 | chloroplast thylakoid | 0 | 0 | 0 |
| GO:0009535 | chloroplast thylakoid membrane | 0 | 0 | 0 |
| GO:0009536 | plastid | 0 | 0 | 0 |
| GO:0009570 | chloroplast stroma | 0 | 0 | 0 |
| GO:0009579 | thylakoid | 0 | 0 | 0 |
| GO:0048046 | apoplast | 0 | 4.2E-41 | 3.21E-16 |
| GO:0005576 | extracellular region | 0 | 2.21E-38 | 1.65E-07 |
| GO:0031977 | thylakoid lumen | 0 | 2.52E-28 | 0 |
| GO:0009295 | nucleoid | 2.9E-24 | 4.65E-23 | 4.92E-20 |
| GO:0015979 | photosynthesis | 5.46E-42 | 2.67E-22 | 0 |
| GO:0010287 | plastoglobule | 5.81E-13 | 7.93E-12 | 3.58E-38 |
| GO:0042742GO:0042830 | defense response to bacterium | 2.51E-24 | 4.6E-09 | 0.025186 |
| GO:0033013 | tetrapyrrole metabolic process | 4.45E-08 | 9.51E-05 | 7.66E-23 |
| GO:0033014 | tetrapyrrole biosynthetic process | 4.45E-08 | 9.51E-05 | 6.95E-19 |
| GO:0006778 | porphyrin metabolic process | 4.45E-08 | 9.51E-05 | 4.05E-22 |
| GO:0006779 | porphyrin biosynthetic process | 4.45E-08 | 9.51E-05 | 1.01E-16 |
| GO:0015995 | chlorophyll biosynthetic process | 0.000139 | 0.000329 | 3.54E-16 |
| GO:0015994 | chlorophyll metabolic process | 0.000139 | 0.000329 | 1.6E-20 |
| GO:0009765 | photosynthesis, light harvesting | 2.49E-10 | 0.002598 | 3.81E-26 |
| GO:0006952GO:0002217GO:0042829 | defense response | 2.44E-13 | 0.003707 | 2.6E-05 |
| GO:0019684 | photosynthesis, light reaction | 4.67E-11 | 0.006023 | 0 |
| GO:0009416 | response to light stimulus | 0.005104 | 0.010097 | 4.63E-16 |
| GO:0009314 | response to radiation | 0.005104 | 0.010097 | 5.67E-15 |
| GO:0051536 | iron-sulfur cluster binding | 4.74E-05 | 0.010097 | 0.01987 |
| GO:0051540 | metal cluster binding | 4.74E-05 | 0.010097 | 0.01987 |
| GO:0009853 | photorespiration | 3.95E-07 | 0.010628 | 0.020344 |
| GO:0051537 | 2 iron, 2 sulfur cluster binding | 8.47E-06 | 0.014976 | 0.036504 |
