# Supplementary material for: A Brassica Exon Array for Whole-Transcript Gene Expression Profiling
Source: PLoS One. 2010 Sep 16;5(9):e12812. doi: 10.1371/journal.pone.0012812 (PMC2940909; doi:10.1371/journal.pone.0012812)
Supplement: Table S2 — Common GO categories over-represented in genes whose transcript abundance is less in leaves compared with roots of Brassica rapa R-o-18 (0.04 MB DOC) [file pone.0012812.s002.doc]

**Table S2** Common GO categories over-represented in genes whose transcript abundance is less in leaves compared with roots of *Brassica rapa* R-o-18

| GO accession | GO Term | BH corrected *p*-value | | |
| --- | --- | --- | --- | --- |
|  |  | 135k Brassica Exon array | 95k Brassica 60-mer array | AtGen Express data |
| GO:0005506 | iron ion binding | 8.18E-13 | 2.30E-18 | 1.76E-05 |
| GO:0020037 | heme binding | 4.12E-13 | 4.16E-14 | 2.38E-07 |
| GO:0046906 | tetrapyrrole binding | 4.12E-13 | 4.16E-14 | 1.52E-05 |
| GO:0004601GO:0016685GO:0016686GO:0016687GO:0016693 | peroxidase activity | 1.85E-18 | 9.41E-14 | 8.21E-14 |
| GO:0016684 | oxidoreductase activity, acting on peroxide as acceptor | 1.85E-18 | 9.41E-14 | 8.21E-14 |
| GO:0016491 | oxidoreductase activity | 3.38E-08 | 3.77E-13 | 2.13E-04 |
| GO:0016209 | antioxidant activity | 3.11E-18 | 2.13E-12 | 1.30E-13 |
| GO:0009055GO:0009053GO:0009054 | electron carrier activity | 1.02E-07 | 1.08E-09 | 1.56E-06 |
| GO:0006979 | response to oxidative stress | 2.72E-12 | 7.23E-09 | 6.76E-05 |
| GO:0042221 | response to chemical stimulus | 3.11E-12 | 1.30E-08 | 2.65E-09 |
| GO:0050896GO:0051869 | response to stimulus | 2.39E-11 | 3.52E-08 | 2.42E-07 |
| GO:0006970 | response to osmotic stress | 9.69E-12 | 2.98E-06 | 1.07E-09 |
| GO:0009651 | response to salt stress | 1.70E-11 | 3.83E-06 | 1.21E-08 |
| GO:0012505 | endomembrane system | 3.16E-12 | 6.58E-06 | 9.00E-04 |
| GO:0006950 | response to stress | 2.25E-10 | 1.79E-05 | 1.68E-05 |
| GO:0009628 | response to abiotic stimulus | 1.21E-08 | 8.25E-05 | 2.30E-04 |
| GO:0046686 | response to cadmium ion | 2.09E-02 | 2.91E-02 | 3.44E-04 |
| GO:0010038 | response to metal ion | 2.25E-02 | 3.25E-02 | 2.28E-05 |
| GO:0010035 | response to inorganic substance | 2.25E-02 | 3.25E-02 | 8.21E-14 |
| GO:0005886GO:0005904 | plasma membrane | 9.53E-11 | 4.40E-02 | 1.58E-17 |
